# Larval ascariasis elicits a prominent IgA and IgG1/2 antibody response to adult *Ascaris* excretory/secretory antigens in pigs

**DOI:** 10.3389/fimmu.2025.1606128

**Published:** 2025-07-30

**Authors:** Zaneta D. Musimbi, Alexandra Laubschat, Larissa Oser, Robert M. Mugo, Benjamin-Florian Hempel, Philipp Höfler, Josephine Schlosser-Brandenburg, Ankur Midha, Sebastian Rausch, Susanne Hartmann

**Affiliations:** ^1^ Center for Infection Medicine, Department of Veterinary Medicine, Institute of Immunology, Freie Universität Berlin, Berlin, Germany; ^2^ Veterinary Center for Resistance Research, Department of Veterinary Medicine, Freie Universität Berlin, Berlin, Germany; ^3^ MF 3 - Experimental Animal Research and 3R - Method Development and Research Infrastructure, Robert Koch Institute, Berlin, Germany

**Keywords:** Ascaris, antibody, IgA, mucosal, systemic, pig, ES products, somatic antigens

## Abstract

Roundworm infections result in morbidity, causing significant health and economic concerns in humans and pigs, respectively. We investigated the humoral responses of *Ascaris suum* infected pigs before and after transition from larval to adult stage and confirmed our previous report on the diagnostic value of human *Ascaris*-specific antibodies. We evaluated the systemic and mucosal humoral responses in *Ascaris* infected pigs at 14- and 35-days post-infection (dpi). *Ascaris*-specific antibodies against larval and adult worm antigens and adult excretory/secretory (ES) products in serum, broncho-alveolar lavage fluid and intestinal mucus were quantified by ELISA. IgA^+^ B cells in jejunal/ileal mesenteric lymph nodes (mLNs) were investigated using flow cytometry. ES products reliably reported parasite-specific IgM, IgA, IgG and IgG1/2 present in sera at 35 dpi (adult stage) and even at 14 dpi (larval stage). Neither variable worm burdens nor the coinfection with *Salmonella* affected the ES-specific antibody profiles. Extracts of the third-stage larvae (L3) were less suited but clearly reported L3-specific secretory IgA in lung and intestine. IgA^+^ B cells expanded in lymph nodes draining jejunum and ileum at day 14 post infection but leveled down to background controls at 35 days after primary infection. A strong correlation between sIgA and eosinophil numbers was seen in the lung, validating previous observations in mice for the definite host. The balanced targeting of L3-somatic antigens and adult ES by sIgA in mucosal sites contrasted with prominent parasite-specific IgA in sera which exclusively reacted to ES products. Collectively, our data indicate extensive antigenic overlap between *Ascaris* life stages, facilitating the detection of pre-patent and larval stage infection. We further point out distinct mucosal/systemic IgA responses in *Ascaris* infection which deserve further functional investigations.

## 1 Introduction

Despite the efforts put in place to curb soil transmitted helminths (STH), an estimated 732 million people as of 2021, are still infected (1). As of 2018, *Ascaris lumbricoides* was described as the most prevalent STH in sub-Saharan Africa (2). The prevalence and intensity of *A. lumbricoides* infection has significantly dropped over the years (1), however, significant morbidity (3, 4) associated with chronic infection still deems it a major public health concern. In parallel, the porcine roundworm *Ascaris suum*, closely related to *A. lumbricoides* (5, 6), is widespread in pigs and ascariasis re-emerged as a veterinary concern in industrialized countries in the context of animal welfare-oriented changes of the animal husbandry (7). *A. suum* impacts the pig industry through liver condemnation and a reduced pig feed conversion ratio resulting in economic losses (8, 9). In addition, *A. suum* bears zoonotic potential (10, 11) thus implicating the pig as a potential reservoir for human ascariasis (12). Markedly, ascariasis may also impact vaccine efficacy against other pathogens (13) and potentially increases susceptibility to coinfections (14).

The life cycle of *Ascaris* in humans and pigs is thought to be similar (15). Upon ingestion of infective eggs, the L3 hatch in the small intestines and breach the cecal-colonic wall (16) to reach the liver. Following further growth in the lung (17, 18), the L3 breaks into the alveolar space and migrates along bronchioles to be coughed up, swallowed and hence relocated back to the small intestine where the larvae molt into L4 (19). The hepato-tracheal migration lasts for roughly 10 days and leads to mechanical injury and larval tissue entrapment that trigger type 2 inflammatory responses involving eosinophils, basophils, alternatively activated macrophages and mast cells that aid in tissue repair (20) in the liver and lung, visible in the white lesions in liver tissue (18, 21–23). While migration has an important role in larval development and host immune evasion (24, 25), it also exposes the host to a repertoire of *Ascaris* stage-specific antigens (21, 23) which results in the induction of humoral responses reflected in serum and mucosal secretions.

*Ascaris*-specific circulatory antibodies (IgG and IgE) are widely used in serological assays to diagnose ascariasis [as reviewed by Roose et al. (26)] and correlate with host protection (21, 27–31). In contrast, mucosal IgA responses [particularly secretory IgA (sIgA)] remain poorly characterized, despite evidence from murine models suggesting an sIgA-mediated defense against migratory larvae in the lungs (30, 31). In humans, IgA exists as two structurally distinct subclasses (IgA1 and IgA2, differing at the hinge region), with IgA1 dominating in serum but both subclasses equally distributed in mucosal tissues (32, 33). However, ethical and logistical constraints have restricted most human studies to serum IgA, leaving mucosal sIgA dynamics in infected mucosal tissues largely unexplored. In experimental animal models, evidence highlights the dual role of intestinal mucus whereby it physically impedes larval invasion (34) while its high sIgA content may also contribute to immune defense against recurrent gastrointestinal helminth infections (reviewed by Ramos et al. (35)]. A focus in mucosal IgA in pigs holds translational significance for both veterinary and human medicine. In pigs, sIgA may represent a key effector mechanism against migratory *A. suum*, as suggested by the high numbers of IgA-bearing cells found in the lungs and small intestinal tissues of *A. suum* infected pigs (21, 36). Further, pigs provide a physiologically relevant model for human infectious diseases (37). Studies have reported IgG responses in pigs and IgG4 responses in humans against *A. suum* lung stage larval lysates (38, 39), leading to successful identification of immunogenic proteins in these lysates, antigen purification and recombinant protein development that would contribute towards serodiagnosis of human and porcine ascariasis (40). Thus, studies on porcine sIgA against *A. suum* may identify conserved protective mechanisms and clinically relevant biomarkers for human ascariasis.

To assess stage-specific antibody profiles, we focused on two key infection stages that together replicate the full *Ascaris* life cycle: larval-stage ascariasis (14 days post infection, dpi), representing the larval migratory phase of infection, and adult-stage ascariasis (35 dpi), representing the intestinal adult phase of the infection. Unlike murine models limited to larval stages or human studies restricted to serum IgA, our approach uniquely captures both tissue-migratory and gut-resident immunity in the natural host. Importantly, our experimental setup allowed us to investigate the specific antibody profiles of pigs infected with *A. suum* (35 dpi) using adult worm ES products which previously displayed diagnostic value in the identification of school children currently infected with *A. lumbricoides* in an earlier study (41) and to test whether these ES products could cross-react with larval-stage antibodies (14 dpi). Overall, our study expands on prior work by testing adult ES antigen’s diagnostic potential across infection phases, comparing mucosal vs. systemic IgA responses and establishing antibody-specific correlates of protection absent in human and/or murine studies. Our findings revealed robust IgM, IgG, IgA responses against adult ES products, distinct targets of secretory versus circulating IgA, and transiently upregulated IgA^+^ B cell expansion in the lymph nodes draining the small intestine of *A. suum* infected pigs.

## 2 Materials and methods

### 2.1 Ethics statement

The experimental studies were implemented at Freie Universität Berlin observing the guidelines stated in the German Animal Welfare Law and the European Convention for the Protection of Vertebrate Animals used for Experimental and other Scientific Purposes. Both studies were ethically approved by the Berlin State Office of Health and Social Affairs (Landesamt für Gesundheit und Soziales; approval numbers G0212/20 and G0107/22) in Berlin (Germany) and are reported in accordance with ARRIVE guidelines 2.0.

### 2.2 Animals and experimental infection

Study 1: A conventional breeder in Brandenburg (Germany) provided a total of 24 six-week-old female hybrid pigs (German landrace and large white). The pigs were allowed to familiarize with the new environment for 30 days prior to the experimental infection. The pigs were randomly assigned into groups based on their body weight. The groups included uninfected controls (n=8), and pigs orally infected with a single dose of 4000 infective *A. suum* eggs (n=16). Food and water were provided *ad libitum*. Animals were housed in indoor stables on wood chips with controlled light, temperature, and ventilation systems. Enrichment was provided in the form of toys and medical training. Pigs were necropsied at 35 days post infection (dpi).

Study 2: Animal details and experimental infection were previously described (42). In brief, animals were categorized into four experimental groups of six pigs (hybrid German landrace and large white, aged 6 weeks and of both sexes) each: uninfected controls, *A. suum* single infection (4 x 2000 infective eggs) and a coinfected group which was first inoculated with 2000 A*. suum* eggs per day for 4 consecutive days, followed by inoculation with 10^7^ CFU of non-typhoidal *Salmonella enterica* Serovar Typhimurium 3 days later (day 7). The 4^th^ group was exposed to *S.* Typhimurium single infection (10^7^ CFU) (42). Pigs were necropsied at 14 dpi (*Ascaris* infection) and 7dpi (*Salmonella* infection).

In both studies, infection status was determined upon arrival and post-infection through fecal egg counts using the Mini-FLOTAC technique (43). Following the standardized protocol from the University of Naples Federico II, individual fecal samples (5.0-5.5 g) were homogenized with saturated saline solution, filtered through a 500 µm sieve, and analyzed in duplicate chambers to ensure accurate quantification.

### 2.3 Sampling

Study 1: Blood was sampled by heart puncture, and bile collected from the gall bladder. Sampling was done on two independent animal experiments. Seven to ten lymph nodes draining the proximal – intermediate jejunum (jejunal mLN) were collected and stored in wash medium (RPMI with 1% fetal calf serum (FCS), 100 U/ml penicillin and 100µg/mL streptomycin; PAN-Biotech) on ice until further processing. Mucus collection protocol was adapted from Krupa et al. (44). Intestinal mucus was isolated from freshly excised small intestinal segments from the most proximal, 10m-long jejunal segment and the most distal, 10m-long ileal segment. The intestinal contents of these segments were flushed out using 0.9% physiological saline. The clean intestines were cut open along the mesenteric border to expose the mucosal epithelium covered with mucous. With a soft-rubber scraper, the mucous was gently removed from the mucosal surface. The remaining section of the intestines was cut open and worms extracted manually due to their macroscopic nature at 35 dpi.

Study 2: Tissue sampling was done as previously described (42). Briefly, blood was collected by heart puncture, gall bladders were collected for bile, bronchoalveolar lavage (BAL) was flushed from the right lung and filtered through a 70 µM strainer. Only blood and bile were sampled from two independent animal experiments. Seven to ten jejunal mLN and three to five lymph nodes draining the ileum (ileal mLN) were collected and stored as stated in study 1.

### 2.4 Antigen preparation

Adult worms were collected from a pig slaughterhouse in Northern Germany. For L3 lysate, female adults were separated from males and washed with 0.9% physiological NaCl. The female worms were cultured overnight in balanced salt solution (HBSS-AB: 127 mM NaCl, 7.5 mM NaHCO_3_, 5 mM KCl, 1 mM CaCl_2_, 1 mM MgCl_2_), 0.1% glucose, 200 U/ml penicillin, 200μg/ml streptomycin, 2.5 μg/ml amphotericin and 50 μg/ml gentamycin (PAN-Biotech GmbH, Germany), the HBSS-AB was changed and culture continued for a period of three days. Shed eggs were collected every 24 hours by collecting the used HBSS-AB and replacing with fresh HBSS-AB. The eggs were then washed and incubated at 30°C for embryonation (45, 46). Fully embryonated eggs were isolated using sucrose step gradient, mechanically hatched using glass beads and processed as previously described (46). Isolated larvae were homogenized using Fastprep-24 (velocity of 4.5 m/s for 30 seconds), centrifuged and the supernatant collected. The supernatant was further sonicated on ice and centrifuged. Supernatant was collected and passed through 0.22 µM sterile filter (Millex-GV, Millipore, Germany).

Adult antigens were prepared as previously described (41). Briefly, whole adult worms were homogenized in liquid nitrogen, sonicated, centrifuged and the supernatant sterile filtered with 0.22 µM sterile filter (Millex-GV). Adult ES products were collected from three worms (1 male, 2 females) cultured in 100 ml of HBSS-AB for two days at 37°C with daily HBSS-AB change. The supernatants were sterile filtered, and the flowthrough concentrated using Vivaspin columns (5 kDa molecular cut-off; Sartorius, Germany). The ES concentrate was further washed in PBS (PAN Biotech, Germany) and filtered (200 nm filter). Protein quantification of all three antigens was done using a microplate reader (Synergy H1, BioTek Germany) and reconstituted to 1mg/ml.

### 2.5 Evaluation of *Ascaris*-specific antibody responses

Indirect ELISA was used to detect *Ascaris*-specific antibodies in serum, bile, BAL fluid, and intestinal mucus. Sample dilutions per antibody and antigen were determined using checkerboard titration (**Table 1**).

**Table 1:** Sample dilutions used for *Ascaris*-specific ELISA.

Antigen	Sample	IgM	IgG	IgA	IgG1	IgG2	sIgA
L3 Lysate	Serum	1:2500	1:2500	1:5	1:100	1:50	–
	Bile	1:50	1:4	1:4	–	–	–
	BAL fluid	–	–	–	–	–	1:20
	Intestinal mucus	–	–	–	–	–	1:10
Adult Lysate	Serum	1:500	1:500	1:5	1:100	1:150	–
	Bile	1:1	1:1	1:1	–	–	–
	BAL fluid	–	–	–	–	–	1:20
	Intestinal mucus	–	–	–	–	–	1:10
Adult ES products	Serum	1:2500	1:2500	1:100			
	Bile	1:4	1:1	1:100	–	–	–
	BAL fluid	1:20	1:10	1:10	–	–	1:50
	Intestinal mucus	–	–	–	–	–	1:20

96-well polystyrene plate (Thermoscientific, *#*442404) were coated with either L3 lysate (20 µg/ml), adult lysate (20 µg/ml) or adult ES products (10 µg/ml) in carbonate buffer (Sigma Aldrich, Germany) at a pH of 9.5 and incubated overnight at 4°C. Plates were subsequently washed three times with 0.05% Tween20 in phosphate-buffered saline (PBS) after every step except for the final wash that was done five times. Blocking of non-specific binding sites was done using 3% BSA in PBS at room temperature (r.t.p) for 1 hour. After washing, samples (either serum, bile, BAL fluid or Intestinal mucus) and standards (two-fold serial dilution of pooled samples of *Ascaris* infected pigs) were added and incubated at r.t.p for 2 hours. Plates were washed and secondary antibodies added. For IgG, Horseradish-peroxidase (HRP) conjugated anti-pig IgG (Sigma-Aldrich, *#*A5670) at a dilution of 1:40000 was used. For IgM, IgA, IgG1 and IgG2, non-conjugated mouse anti-pig IgM (Bio-Rad, *#*MCA637GA) diluted at 1:20000, mouse anti-pig IgA (Bio-Rad, *#*MCA638GA) diluted at 1:1000, mouse anti-pig IgG1 (Bio-Rad, *#*MCA635GA) diluted at 1:1000 and mouse anti-pig IgG2 (Bio-Rad, *#*MCA638GA) diluted at 1:1000, mouse anti-pig sIgA (Bio-Rad, *#*MCA634GA) diluted at 1:10000 was used. After 1 hour incubation at r.t.p, HRP-conjugated goat anti-mouse IgG (Bio-Rad, *#*STAR117P) at a dilution of 1:10000 was added to plates previously added with non-conjugated antibodies. Final wash was done, 3,3’,5,5’-Tetramethylbenzidine (TMB) substrate (Invitrogen, *#*00-4201-56) was added and allowed to develop in the dark for 20 minutes. Sulphuric acid (H_2_SO_4_) (Sigma Aldrich) was used to stop the reaction and optical density measured at 450 nm and 570 nm (reference wavelength).

Background was accounted for by subtracting blank wells followed by subtraction of the reference wavelength (570 nm). Top standards were concentrated twice as much as the diluted sample and further assigned an arbitrary ELISA unit (AEU) of two. Standard curve was generated, and the sample concentration determined.

### 2.6 Preparation of cell suspension and flow cytometric immunophenotyping

For the isolation of jejunal and ileal mLN lymphocytes, excessive fat and connective tissues were removed from the mLNs. Lymph nodes from each section (ileal or jejunal) were pooled, minced and forced through a 70 µM strainer. The isolated cells were then pelleted and resuspended in complete Iscove’s Modified Dulbecco’s Medium (IMDM (PAN-Biotech GmbH, Germany) supplemented with 10% heat-inactivated fetal calf serum, 1% penicillin/streptomycin). For immunophenotyping, lymphocytes were plated at 3 x 10^6^ cells per well in a conical 96-well plate. Surface and intracellular markers were stained following standard protocols (47) with the antibodies listed in **Supplementary Table 1**. Cells were acquired on a FACS ARIA III (BD Biosciences) and analyzed with FlowJo version 10 (Tree Star).

### 2.7 Statistical analysis

Normality and homogeneity were tested using Shapiro-Wilk’s test and Levene’s test respectively. Group comparisons were done using Kruskal Wallis test followed by Wilcoxon test or Games Howell test. Benjamin Hochberg adjustment was done in cases of multiple comparison. Correlation analysis was done using either spearman rank or Pearsons Linear Regression. Statistical analysis and visualization were done in R (48) (v4.4.0; 2024-04-24).

## 3 Results

### 3.1 Robust responses to ES products of adult worms in pre-patent *A. suum* infection (35 dpi)

**Figure 1:**
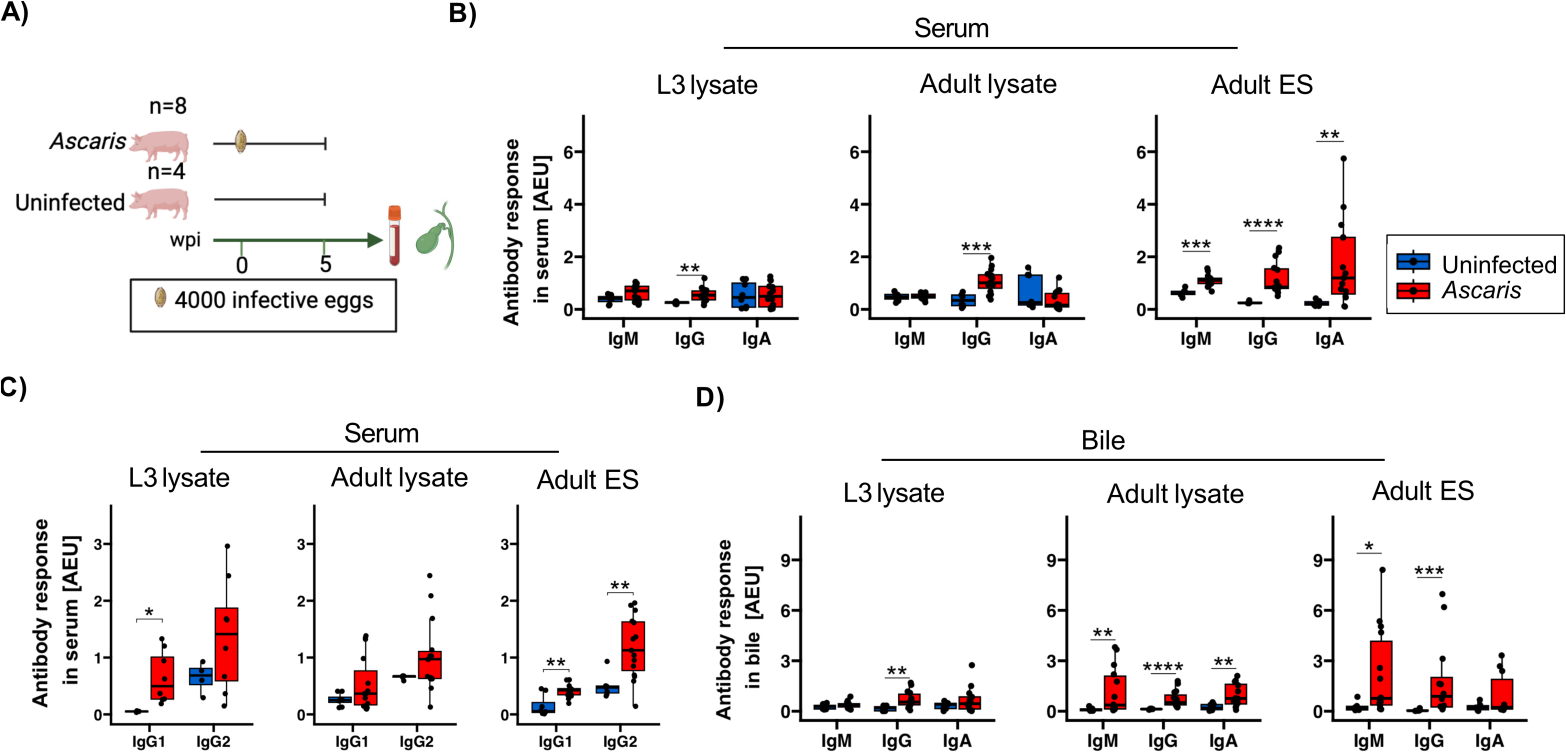
Antibody isotype responses against *A*. *suum* L3, adult and adult ES antigens in serum and bile 35 dpi. **(A)** Schematic illustrating experimental infection of the pigs. Eight pigs were infected with a single dose of 4000 infective *A*. *suum* eggs. In parallel, four uninfected controls were included. Box plots illustrating median serum **(B)** IgM, IgG, IgA and **(C)** IgG1 and IgG2 subclass responses against *A*. *suum* L3 lysate, adult lysate and adult ES products. **(D)** Box plots illustrating median bile IgM, IgG and IgA responses against *A*. *suum* L3 lysate, adult lysate and adult ES products. The data is reported in arbitrary ELISA units (AEU). Whiskers indicate 95% percentile. Significance assessed using the Wilcoxon test and depicted by p<0.05: *p<0.01: **p<0.001: ***p<0.0001: ****. *Ascaris*-infected n= 16 and uninfected controls n= 8.

As a gold standard, serological ELISAs are used to determine antibody responses during *Ascaris* infection with IgG as the widely investigated *Ascaris*-specific antibody in sera (26). We have recently shown that antibody serology based on the recognition of adult *Ascaris* excretory/secretory products (ES) products (and not lysates comprising somatic antigen of L3 and adult worms) reliably determined the *Ascaris* infection status of school children in an *A. lumbricoides* endemic region in Western Kenya (41). Here, we adapted the protocol for the survey of humoral responses of pigs that had been inoculated with 4,000 *A. suum* eggs 35 days earlier (**Figure 1A**) and compared the responses to ES products of adult worms, lysates of egg-derived L3 and lysates prepared from adult *A. suum*.

IgM, IgG and IgA present in sera of the infected group clearly reacted with adult ES, whereas only IgG responded to the somatic antigens present in lysates of L3 and adult worms (**Figure 1B**). To evaluate if the IgG responses held further information on the type-1 or -2 like immune activity during B cell priming, we further assessed the IgG subclass composition, focusing on IgG1 supported by IL-4 signaling and IgG2 promoted by IFN-γ signals (**Figure 1C**). In accordance with the strong type-2 inducing activity associated with tissue damage during larval migration, we determined a significant IgG1 response to the somatic antigens of the tissue-migratory L3 stage in all infected animals, paralleled by more variable IgG2 responses. Interestingly, other IgG isotypes appeared to be accountable for the elevated IgG responses to somatic antigens of adult worms, as neither IgG1 nor IgG2 responded to the adult somatic antigens in a consistent manner (**Figures 1B, C**). In addition, the ES products of adult worms elicited a third pattern concerning type1/2 associated antibodies, composed of significant IgG1 as well as IgG2 responses in sera of infected pigs (**Figure 1C**).

Bile comprises all major subclasses of antibodies and biliary epithelial cells of both humans and pigs express the polymeric IgA receptor required for transport and release of sIgA. Different from rodents where biliary IgA largely derives from blood, sIgA in human and pig bile primarily derives from plasma cells localized along the biliary tree (49). We hence investigated antibody responses in bile and found exclusive and significant IgG binding to L3 antigens, reflecting the pattern seen in serum (**Figure 1D**). Similarly, the ES products of adult worms mirrored the responses in serum, exhibiting significant binding of IgM and IgG. However, the low responses of sIgA to adult ES products in bile differed from robust ES specific responses seen for monomeric IgA in the sera of infected pigs, indicating that sIgA specific for adult ES was not a prominent component of biliary excretions. A rather unexpected observation concerned the isotype responses to adult worm somatic antigens, with IgM as well as IgA exhibiting significant antigen binding in bile, but no in serum.

Together, the strong humoral responses of *A. suum* infected pigs composed of IgM, IgA and type1/2 associated IgG isotypes confirmed our previous observation of a broad range of isotype responses generated against the *Ascaris* ES products of roundworm infected humans (41). The IgG1 binding to L3 antigens seen in all infected pigs indicated a significant Th2 bias of the environment experienced by L3-responsive B cells during class switching. However, some individuals simultaneously displayed significant IgG2 responses to the migratory L3, possibly indicating variation in the IFN-γ competence of tissues passed by the L3 in individual pigs.

### 3.2 Differential expulsion of *A. suum* does not correlate with ES-specific antibody profiles

**Figure 2:**
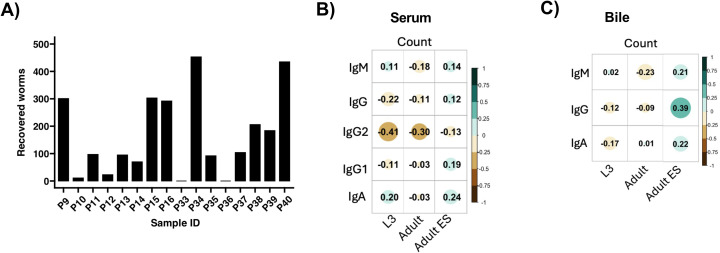
Correlation between antibody responses and worms recovered from the intestines. **(A)** Bar plot depicting the number of worms recovered from the intestines of 16 pigs infected with a single dose of 4000 infective *A*. *suum* eggs at 35 dpi. Correlation plots illustrating the association between worm count and specific antibody responses in the serum **(B)** and bile **(C)**. Spearman’s rank correlation was used. Color gradient from brown to blue-green indicate negative to positive correlation. Circle size increase with increase in correlation coefficient.

Next, we asked whether the significant *Ascaris*-specific antibody responses in both serum and bile were associated with the worm burden, or, conversely, with the early expulsion of the L4 stage known to typically occur between day 14 and 21 after infection (50). Intestinal worm recovery revealed a highly variable worm distribution within the 16 infected pigs, of which two had expelled all worms (**Figure 2A**). As reported earlier for naturally and trickle infected pigs (51, 52), the antibody responses in serum did not correlate with the worm burden of the single dose experimentally infected pigs (**Figure 2B**). Similarly, the levels of *Ascaris*-specific antibodies in bile were not linked with the worm burden (**Figure 2C**).

### 3.3 Strong cross-reactive antibody responses to adult ES at 14 days post infection are not altered by *Salmonella* coinfection

**Figure 3:**
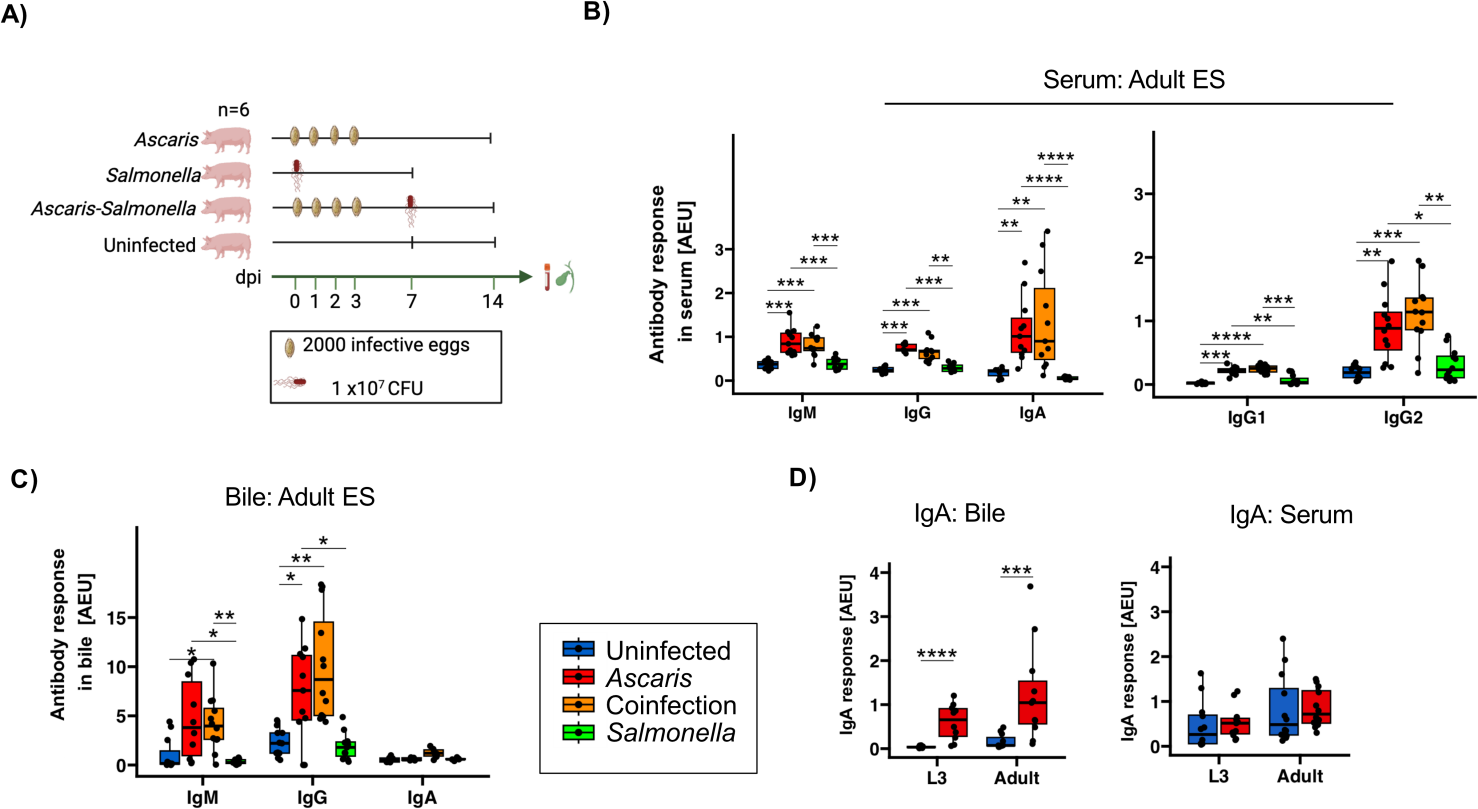
Comparable *Ascaris*-specific antibody responses in *Ascaris* single- and co-infected pigs. **(A)** Schematic illustrating experimental infection of the pigs. Box plots illustrating median serum IgM, IgG, IgA, IgG1 and IgG2 responses **(B)** and median bile IgM, IgG and IgA responses **(C)** against *A*. *suum* adult ES products. **(D)** Box plots illustrating L3- and adult-specific IgA responses in bile and serum. Blue, red, orange and green represent uninfected controls, *A. suum* infected, *A*. *suum- S*. Typhimurium coinfected and *S.* Typhimurium infected pigs respectively. Whiskers indicate 95% percentile. Significance is represented by p<0.05: *p<0.01: **p<0.001: ***p<0.0001: ****. Significance was tested using Kruskal Wallis with Dunn or Games-Howell test and Wilcoxon test. Uninfected controls n = 12, *Ascaris* single infected n = 12, *Ascaris-Salmonella* coinfected n = 12, *Salmonella* single infected n =12.

*Ascaris-Salmonella* coinfection is highly likely in helminth endemic regions and the pig husbandry (14). The control of *Salmonella* requires Th1 cells (53), but the microbe may evade immune pressures in acquiring a dormant stage in infected pigs, posing a long-term threat to human health (54). We recently showed that a concurrent *Ascaris* infection impairs the control of *Salmonella* in coinfected pigs (42). As recent work by others showed that secondary *S.* Typhimurium infection may disrupt pre-existing pathogen-specific antibody responses (55), we went back to the material collected from the trial cited above (42) and investigated the antibody profiles in *Ascaris* single-infected pigs at day 14 dpi compared to pigs exposed to *Salmonella* coinfection at day 7 of the ongoing *Ascaris* infection (**Figure 3A**).

First, we assessed the L3 induced humoral responses for cross-reactivity with adult worm derived ES products sera based on day 14 after infection. Like at 35 dpi, IgM, IgG, IgA as well as IgG1/IgG2 responses were significantly elevated in *Ascaris*-single infected pigs in comparison to uninfected controls (**Figure 3B**). Similarly, the antibody profiles in bile collected at day 14 reflected the responses seen at day 35, including the absence of significant IgA responses reactive to adult ES (**Figure 3C**). Conversely, a closer look into L3 and adult specific IgA revealed a significant increase in bile and not serum (**Figure 3D**).

Based on the responses to adult ES, neither IgM, IgA, total IgG or the IgG1/2 subclass responses differed between the *Ascaris* single and coinfected groups, indicating that the responses against antigens shared between larval and adult worm stages were not altered by the co-exposure to *Salmonella* infection during larval stage *Ascaris* infection (**Figure 3C**). Similarly, the coinfection had no impact on the antibody response against adult ES in bile, as we detected comparable ES-specific IgM and IgG responses in *Ascaris* single and coinfected pigs at 14 dpi (**Figure 3C**).

Together, the strong binding of day 14 antibodies to ES products of adult worms illustrated the large overlap in the antigenic nature between larval and adult *Ascaris* stages, with mass spectrometry confirming 39.7% shared protein composition (**Supplementary Figure 1**; **Supplementary Material 2**). Furthermore, the similar profiles seen in *Ascaris* single- and co-infected pigs underline the robustness of the isotype profiles generated against antigens shared by the different worm stages.

### 3.4 *Ascaris*-specific sIgA strongly associates with eosinophil influx in BAL

**Figure 4:**
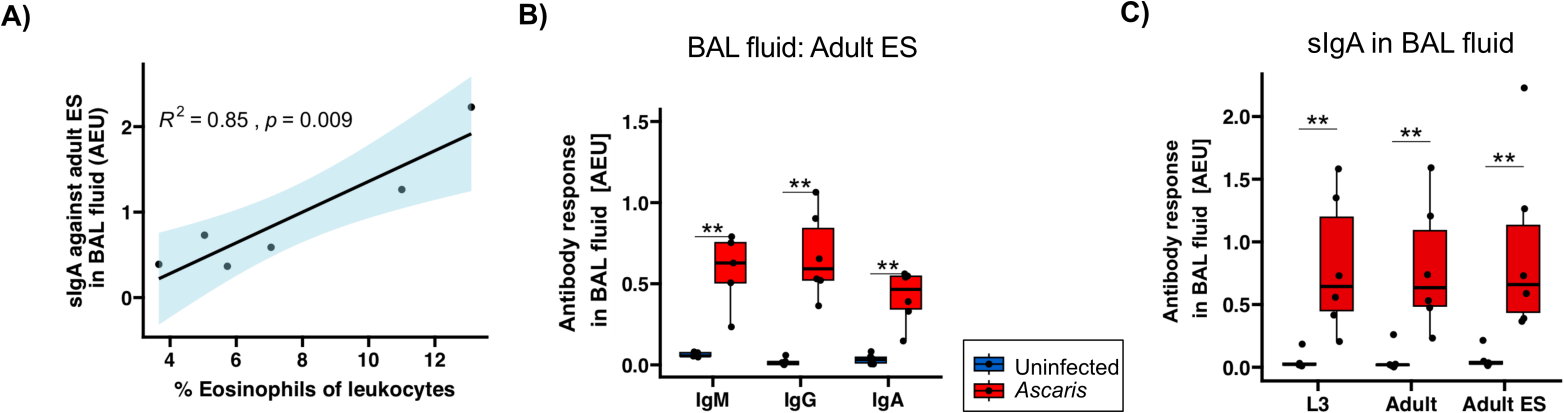
Strong positive correlation between sIgA against *A*. *suum* adult ES and eosinophil influx. **(A)** Pearson correlation of sIgA against adult ES products and %eosinophil frequencies of leukocytes in BAL (42). **(B)** Box plots illustrating median IgM, IgG and IgA responses against *A*. *suum* adult ES products in BAL fluid. **(C)** Box plots illustrating sIgA against L3 lysate, adult lysate and adult ES in BAL fluid. Blue and red denotes uninfected controls and *A. suum* infected pigs, respectively. Whiskers indicate 95% percentile. Significance determined by Wilcoxon test is represented by p<0.05: *p<0.01: **.

Recent work in the semi-permissive mouse model reported a correlation between eosinophil and sIgA responses in the lung of *Ascaris* L3-infected mice which was important for the efficient control of the larval stage (30, 31). We therefore surveyed sIgA and eosinophil frequencies in the BAL at 14 dpi, hence shortly after completion of body migration to see whether a similar correlation existed in the definite pig host of *A. suum*. Indeed, the elevated eosinophil frequencies determined in infected pigs (7.59 ± 3.68 at day 14; 1.02 ± 0.60 in uninfected controls) (42) strongly correlated with the levels of sIgA specific for adult ES products (**Figure 4A**) and L3/adult somatic antigens (**Supplementary Figure 2**).

In addition to the robust *Ascaris*-specific IgA responses, we also found significant IgM and IgG responses against adult ES in the BAL (**Figure 4B**). Importantly, a survey of sIgA reactivity with L3 and adult stage somatic antigen resulted in similarly high responses as seen for adult ES products (**Figure 4C**). Hence, L3 somatic antigens, likely comprising tegument-derived antigenic surface structures as well as internal antigens that may be released by L3 trapped in small intestine, liver or lung tissue, elicited an sIgA response detectable in the lung. This extended to adult lysate, indicating cross-reactivity of the antibodies against L3 with adult worm somatic antigens, as these pigs were dissected before the transition of the larval to the adult stage.

Overall, our data show a clearly exhibited anti-*Ascaris* adult ES antibody responses at 14 dpi in the lung. A positive association between sIgA responses and eosinophil influx detected in the definite pig host confirmed findings in the murine *Ascaris* model. Further, we found strong sIgA responses directed against L3 stage antigens in the lung.

### 3.5 Strong IgA^+^ B cell expansion in ileal compared to jejunal MLN during acute larval stage infection

**Figure 5:**
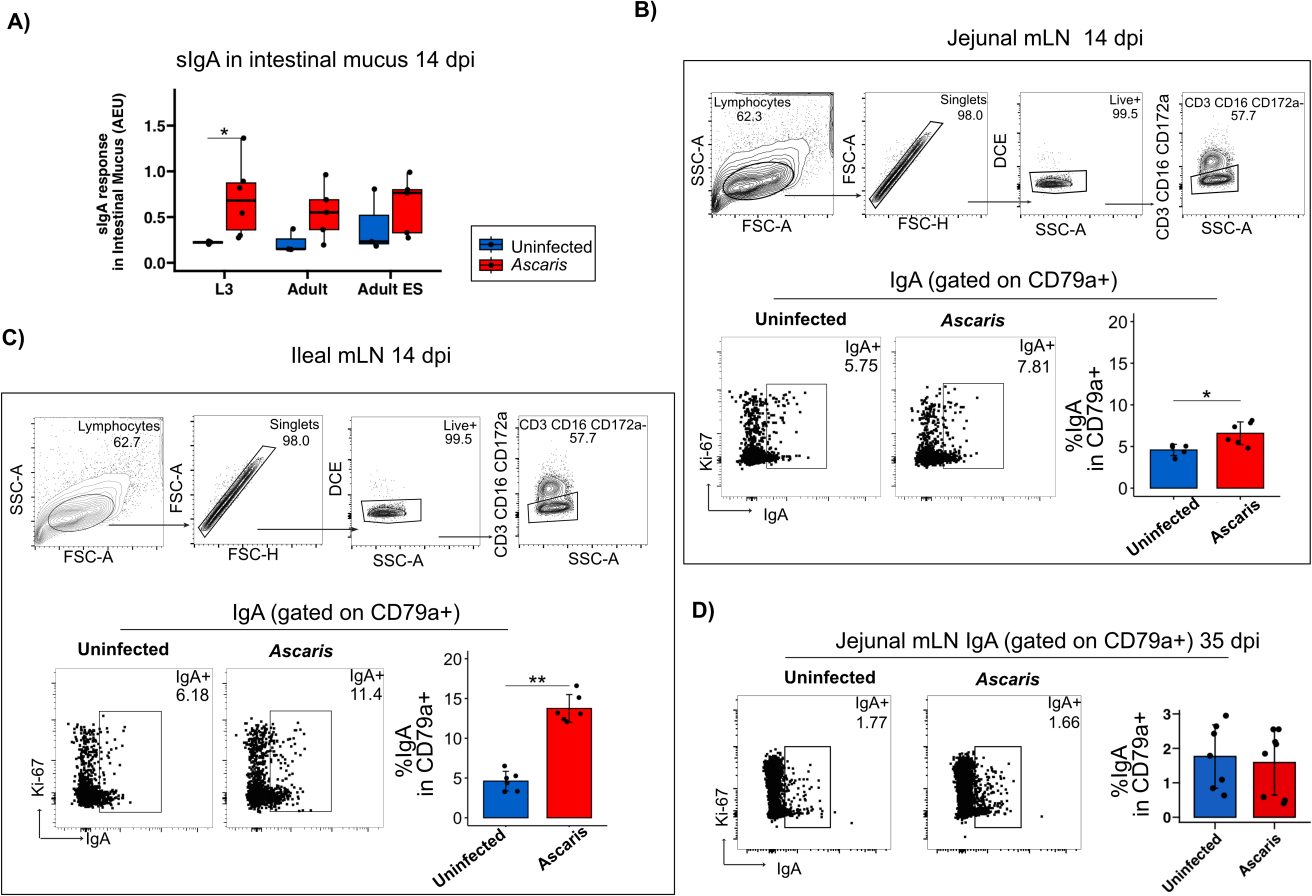
IgA^+^ B cells are highly induced in the ileal mLN 14 dpi. **(A)** Boxplots of sIgA against the three antigens in intestinal mucus. Exemplary flow cytometry plots depicting the gating strategy used to identify CD79a^+^ B cell populations in mLNs. Representative flow cytometry plots of IgA^+^ cells gated on CD79a^+^ cells and bar plots of mean frequencies of % IgA producing B cells in jejunal **(B)** and ileal **(C)** mLN 14 dpi. **(D)** Representative flow cytometry plots of IgA^+^ cells gated on CD79a^+^ cells and bar plots of mean frequencies of % IgA producing B cells in jejunal mLN 35 dpi. Bar plot error bars indicate mean sd. Boxplot whiskers indicate 95% percentile. Significance determined by Wilcoxon test is represented by p<0.05: *p<0.01: **.

Following the detection of prominent sIgA responses directed against somatic antigens and ES products of *A. suum* in the lung, we next assessed the sIgA responses in the small intestinal mucus at 14 dpi. Interestingly, L3 stage somatic antigens were giving the clearest results with significantly elevated sIgA responses in infected pigs, mirrored in trend by the recognition of adult worm extracts and, by a weak trend, in the response to adult ES products (**Figure 5A** **).** Hence, somatic antigens of larval stage undergoing body migration were met with significant sIgA responses in lung and intestine of infected pigs, which apparently cross-reacted with adult worm antigens despite the lack of contact with adult worms in the investigated pigs. Thereby, both somatic/tegument structures shared by larval and adult worms as well as ES products shared between the stages were targeted by significant antibody responses along the course of *Ascaris* infection.

Roepstorff et al. followed *A. suum* single-dose infections and detected larvae in the small intestines (SI) as early as 7 dpi with a peak at 14 dpi. Further following the localization of L4 in narrow time slots (10-, 14-,17-,21- and 28 dpi), the group demonstrated the remarkable shifts of the L4 stage from the proximal towards the distal SI in the process of early expulsion, which removes most of the larvae shortly after their return to the small intestine (50). Although B cells undergoing class-switch recombination in Peyer’s patches and isolated lymphoid follicles may migrate to the gut-draining MLNs for further differentiation before entering the circulation, local IgA induction also occurs in the MLN (56). To see whether IgA promoting signals also extended to the MLN during *Ascari*s infection, we assessed the proportions of IgA^+^ B cells in the LN draining the mid small intestine (jejunum) compared to ileal LN draining the terminal SI (**Figures 5B, C**; gating strategy of CD79a provided in **Supplementary Figure 3**). Indeed, IgA^+^ B cells were significantly increased in the jejunum draining MLN of day 14 infected pigs (**Figure 5B**, several jejunal LN were pooled per individual**).** Strikingly, the IgA response associated with the infection was far stronger in ileal LN (**Figure 5C** **).** Furthermore, B cells derived from the jejunum MLN at day 35 post infection did not comprise higher proportions of IgA class switched B cells anymore (**Figure 5D**), indicating that the IgA responses seen at day 14 ceased within the following weeks in a primary infection.

In conclusion, acute *Ascaris* infection resulted in significantly elevated IgA^+^ B cell expansion in the LN draining the mid and distal small intestine, with a more pronounced response observed in ileal LN. This stronger response in the ileal LN may be linked to the initial tissue invasion by hatched L3 larvae, which reportedly occurs in the distal SI next to cecum and colon (57). Alternatively, the robust IgA response seen in the distal LN might indicate responses to ES products released by the larval stages, which are shifted distally during early expulsion, potentially triggering stronger responses in ileal compared to jejunum LN. Additionally, the repeated inoculation of pigs with four doses of 4000 eggs over four consecutive days may have accelerated the distal migration of the first L4 larvae, possibly due to enhanced type 2 immune activity in the small intestine induced by subsequent infections. Further studies are needed to clarify these mechanisms and establish a definitive explanation for the observed regional differences in IgA responses.

## 4 Discussion

In this study, we demonstrate the effectiveness of the nematode ES products of adult A. *suum* worms for immunodetection and identified seroconversion starting at 14 dpi, occurring both in the serum and at mucosal sites of *A. suum*-infected pigs. A combined IgG1/IgG2 response indicated the involvement of Th2 and Th1 cellular responses in shaping antibody production against larval ascariasis. In addition, *Ascaris*-specific IgA production was prominent in the lungs and intestines and associated with elevated eosinophil counts in the lung.

Natural *A. lumbricoides* infections in humans are known to induce antibodies across all isotypes (IgM, IgG, IgA, IgE) (58). In the current study, however, we did not detect significant levels of IgM, IgA and IgG2 against lysates of L3 or adult worms in the serum at 35 dpi. In contrast, infected pigs exhibited significant ES-specific IgM, IgG, IgA, IgG1 and IgG2 responses in serum. These findings align with our previous work, which detected anti-*Ascaris* IgM, IgG, IgA, IgE, and IgG subclasses 1–4 against adult ES but not somatic L3 or adult antigens in *A. lumbricoides*-infected children (41). Consistent with other experimental pig studies which investigated the antibody responses to single-dose and weekly trickle-infections (21, 27, 59), the current study reports seroconversion already at 14 dpi. Specifically, we show that sera collected from *A. suum* infected pigs at 14 dpi, shortly after the completion of L3 to L4 transition, respond to ES products of adult worms and display similar magnitude and isotype composition as the adult ES-specific antibodies seen in pigs at d35 (about 10 days after the L4 to adult transition). We, thus, achieved a main objective of the study by demonstrating that ES products, readily available from adult worms, are suitable for the reliable detection of seroconversion approximately two weeks before the transition from L4 to adult worms. Collectively, the findings signified the reliability of the adult ES antigen for immunodetection during both early and mid-stage experimental porcine ascariasis. The study prioritized readily available diagnostic materials, including adult ES products, egg-stage L3 antigens, and adult worm somatic antigens, all of which can be produced in larger quantities. We found that adult ES products contain a unique protein composition (6.4% distinct from L3 and adult somatic antigens) including proteins such as neprilysin, maltase glucoamylase, sucrase-isomaltose and venom allergen 3. Importantly, Wang et al. showed an overlap of these proteins in L3-lung and L4 ES and not ES derived from egg-stage L3 (60). Whether or not they contribute to the serological diagnostic potential of adult ES antigens against larval stage ascariasis remains to be determined. Nevertheless, the similarity in composition supports the specificity of adult ES antigens for detecting migratory larval stage-specific antibody responses.

Typically, cytokines skew immunoglobulin isotype selection in B cells. Gastrointestinal helminths induce a mixed immune response (Th1, Th2) with predominant type 2 immunity involving Th2 cytokines IL-4 and IL-5 (61–63) that provides protective immunity (58, 64). These Th2 cytokines are further reported to influence IgG1, IgE and IgA antibody production (65–67). In contrast, IgG2 levels are supported by Th1 cytokines IFN-γ and IL-12 (68, 69). Thus, IgG subclasses, IgG2 and IgG1, are generally described to be markers of Th1 and Th2 cellular responses respectively (70). Our data revealed a mixed IgG2/IgG1 systemic humoral response at 14- and 35 dpi, consistent with the previously described Th1/Th2 systemic cellular response (17). Corroborating this outcome, pigs immunized with fractions of *A. suum* adult antigen (28) and mice immunized with crude extract of either the adult worm, adult worm cuticle or infective larvae (29) exhibited high levels of specific IgG1 and IgG2. In addition, we and Cooper et al. (41, 58) have previously shown mixed specific IgG subclass responses in humans naturally infected with *A. lumbricoides*. Studies on *Heligmosomoides polygyrus bakeri (H. polygyrus)* showed that IgG subclasses 1, 2a/c and 3 activate granuloma macrophages promoting larval adherence and immobilization (71, 72). Therefore, the observed IgG1/IgG2 response in larval ascariasis may similarly contribute to macrophage activation facilitating larval adherence and immobilization.

Further, we show elevated levels of *Ascaris*-specific IgM, IgG and IgA in the BAL fluid 14 dpi: indicative of an early anti-*Ascaris* humoral response in the lower respiratory tract during larval tissue migration. Additionally, BAL fluid and intestinal mucus had elevated levels of *Ascaris*-specific sIgA. In the gut, eosinophils are essential for IgA synthesis through both T cell dependent and independent mechanisms (73, 74), regulating sIgA production (75). Comparing *Ascaris*-infected wildtype and eosinophil-deficient mice, Nogueira et al. (31) demonstrated the eosinophil dependence of general and *Ascaris*-specific sIgA production in the lung. The authors further showed that increased sIgA levels positively correlated with eosinophil influx and negatively correlated with larval burden depicting a network involving innate and humoral immune responses that control the parasite burden during *A. suum* infection in mice (31). We did not quantify the number of lung larvae as we dissected pigs earliest at day 14, about one week after lung passage of L3. However, we found a strong positive correlation between eosinophil accumulation and *Ascaris*-specific sIgA in BAL fluid at day 14. IL-5 supports the survival of eosinophils but also promotes the antibody secretion and maintenance of IgA^+^ mucosal plasma cells. We recently reported peak eosinophilia in the BAL fluid of *Ascaris* infected pigs at day 14 post infection, followed by a rapid decline (17, 42). However, coinciding with the maintenance of Th2 cells in the lung for several weeks, eosinophilia in lung tissue remained stable for at least 5 weeks post infection (17). Furthermore, earlier work reported repressed IL-5 expression in the porcine lung during L3 passage, followed by a strong upregulation at day 14 and the maintenance of elevated IL-5 expression until at least 4 weeks post infection (13, 76). It is hence conceivable that local IL-5 sources, eosinophils and plasma cells create conditions hampering L3 passage through the porcine lung, similar to the recently reported findings in mice. More work is required to see if an IL5-eosinophils-IgA axis may curb *Ascaris* (re-)infection and whether this precedes the development of a pre-hepatic intestinal immune barrier.

We further investigated IgA producing B cells in mLNs and found increased levels of IgA^+^ B cells in both ileal and jejunal mLNs 14 dpi. Distinctively higher levels of IgA^+^ cells were demonstrated in the mLN of the ileum in comparison to jejunum. *Ascaris* larvae return to the small intestines as early as 7 dpi, peaking at 14 dpi (50). Elevated levels of IgA antibody secreting cells in the distal part of the jejunum 10 dpi have been associated with the early immune response against antigens of penetrating larvae (57). Alternatively, the shift of the larval stages towards the distal small intestine following completion of body migration due to the onset of expulsion mechanisms may result in the high availability of worm products in the mLN draining the ileum and contribute to the elevated IgA class switch. However, we acknowledge that dietary antigens could also influence regional IgA responses, as food-derived stimuli may differentially prime immune activity along the intestinal tract. While our findings support a strong infection-driven IgA response, future work should dissect potential interactions between diet and *Ascaris*-induced immunity by profiling antigen-specific IgA+ cells in PP and mLNs under controlled dietary conditions. Additionally, future studies will include the investigation of IgA responses in the porcine Peyer’s patches as well as the quantification of *Ascaris*-specific IgA^+^ cells in PP and in the lymph nodes draining lung and intestine.

An intriguing finding of this study is the functional systemic humoral response observed during coinfection with *S.* Typhimurium. Secondary infection with *S*. Typhimurium after primary *Plasmodium yoelii* or influenza A infection resulted in abrogated primary pathogen-specific antibody production (55). In contrast, the present work demonstrated comparable *Ascaris-*specific circulatory antibody responses in *Ascaris* single- and coinfected pigs. In agreement with our observation, mice coinfected with *Salmonella* and the strictly enteric *H. polygyrus* exhibited a strong Th2-biased circulatory IgG1 response (77). While *A. suum* and *H. polygyrus* induce Th2 immune responses, *P. yoelii* or influenza induce Th1 immune responses. Furthermore, monocytes, which are linked to an inflammatory gene expression profile, have been implicated in germinal center disruption (55). Primary infection with *Ascaris* inhibited the activation and recruitment of inflammatory blood monocytes in coinfected pigs (42), providing a possible link to the observed functional systemic humoral response. These findings suggest that abrogation of pre-existing antibody repertoires is only possible in the case of an existing inflammatory Th1 phenotype. Since this was beyond the scope of our study, no further investigation into the topic was done.

While natural *A. lumbricoides* infections in humans are known to induce IgE responses (41, 58), this study did not quantify *Ascaris*-specific IgE in experimentally infected pigs due to technical and methodological constraints. A primary limitation was the lack of commercially available, standardized anti-porcine IgE monoclonal antibodies required for reliable antigen-specific ELISA detection, as existing reagents for porcine IgE assays remain underdeveloped compared to human and mouse IgE tools (78–81). Despite the established role of IgE in IL-4-mediated anti-helminth immunity (82, 83) and protection against reinfection (84), the Th2-associated IgG1 response served as a functional surrogate, reflecting similar immune pathways.

To conclude, our study indicates strong systemic and mucosal antibody responses in pigs as early as 14 dpi, highlighting the diagnostic potential of adult ES products for detecting early-stage ascariasis. The mixed IgG1/IgG2 response and prominent mucosal IgA production underscore the complexity of the immune response to *Ascaris*, which likely involves both Th1 and Th2 pathways. The positive correlation between eosinophil infiltration and sIgA levels in the lungs may suggest a role for eosinophil/IgA in resistance to larval migration in reinfections. The initiated systemic humoral response induced by *Ascaris* was unperturbed in the presence of an enteric bacterial coinfection. Future studies should focus on identifying specific ES proteins responsible for immune recognition and protection, as well as clarifying the role of serological versus mucosal IgA in pigs.

## Data Availability

The datasets presented in this study can be found in online repositories. The names of the repository/repositories and accession number(s) can be found in the article/**Supplementary Material**.
