# Supplementary material for: Larval ascariasis elicits a prominent IgA and IgG1/2 antibody response to adult Ascaris excretory/secretory antigens in pigs
Source: Front Immunol. 2025 Jul 30;16:1606128. doi: 10.3389/fimmu.2025.1606128 (PMC12343609; doi:10.3389/fimmu.2025.1606128)
Supplement: Supplementary file 2 [file Table1.docx]

Supplementary Material

# Supplementary materials and methods

**Proteomic Profiling of *Ascaris* Antigens**

Antigens (*Ascaris* larval and adult worm lysates and ES products) were lysed using lysis buffer (6M urea in 50 mM ammonium bicarbonate pH 8.5 supplemented with 5 mM DTT) followed by heating at 60 °C for 30 min at 1000 rpm, then alkylated using iodoacetamide (IAA, final 20 mM) for 30 min in the dark at room temperature (r.t.p). The reaction was quenched by adding DTT (final 5mM) and incubated for 15 min at r.t.p. Subsequently, the urea concentration was reduced to ≤ 1M by diluting the samples with 50 mM ammonium bicarbonate (ABC) buffer. Proteolytic digestion was performed by adding trypsin/rLysC mix (1:50 w/w enzyme to protein concentration, Promega) and incubated overnight at 37 °C (at 1000rpm) in a ThermoMixer (Eppendorf). To ensure complete digestion, an additional amount of trypsin (1:100 w/w) was added after 4 hours. Digestion was stopped by acidifying the samples with formic acid (FA) to a final concentration of 1% and recovered peptides were desalted using a ZipTip C18 (Thermo Fisher Scientific Inc.) following the manufacturer’s instructions. The eluates were vacuum concentrated (Eppendorf Concentrator 5301, Eppendorf AG, Germany) and reconstituted separately in 20 µL 0.1% FA and 4% acetonitrile (ACN).

From this 2 µg were injected into a nano liquid chromatography (nHPLC) system (Dionex UltiMate 3000, Thermo Fisher Scientific Inc.) coupled to an QExactive ultrahigh-resolution mass spectrometer (Thermo Fisher Scientific Inc.). The peptide mixture was concentrated onto an Acclaim PepMap100 C18 trap column (3 µm, 100 Å, 75 µm inner diameter, Thermo Fisher Scientific Inc.) and subsequent fractionated on an Acclaim PepMap100 C18 capillary column (2 µm, 100 Å, 75 µm inner diameter, Thermo Fisher Scientific Inc.) at an eluent flow rate of 300 nL/min. Mobile phase A consisted of 0.1% (v/v) formic acid in water, while mobile phase B contained 0.1% (v/v) formic acid and 80% (v/v) ACN in water. The column was pre-equilibrated with 5% mobile phase B, followed by an increase to 44% mobile phase B over 100 minutes. Mass spectra were acquired in a data-dependent mode, utilizing a single MS survey scan (*m/z* 350–1650) with a resolution of 60,000, and MS/MS scans of the 15 most intense precursor ions with a resolution of 15,000. The dynamic exclusion time was set to 20 seconds, and the automatic gain control was set to 3 × 10^6^ and 1 × 10^5^ for MS and MS/MS scans, respectively.

Acquired nLC-MS/MS spectra as mascot generic files (.mgf) were matched to the UniProt (Taxon ID: 6251, *Ascaris suum*) reference proteome using MaxQuant (1) software (version 2.3.1.0). Generic settings were set to a significance threshold of protein FDR ≤ 0.01 and the settings for trypsin as the proteolytic enzyme; a maximum of two miss cleavages; 5.0 ppm peptide tolerance; peptide charges of ≥ 2+; variable modifications: oxidation, deamidation, and acetylation (protein N-terminus) and fixed modification: carbamidomethyl; 20 ppm MS/MS tolerance to identify the corresponding protein.

Mass spectrometry proteomics (raw, mzML, .txt) data have been deposited with the ProteomeXchange Consortium (2) via the MassIVE partner repository with the data set identifier PXD065260.

1. Cox J, Mann M. MaxQuant enables high peptide identification rates, individualized p.p.b.-range mass accuracies and proteome-wide protein quantification. *Nat Biotechnol* (2008) 26:1367–1372. doi: 10.1038/NBT.1511

2. Vizcaíno JA, Deutsch EW, Wang R, Csordas A, Reisinger F, Ríos D, Dianes JA, Sun Z, Farrah T, Bandeira N, et al. ProteomeXchange provides globally coordinated proteomics data submission and dissemination. *Nat Biotechnol* (2014) 32:223–226. doi: 10.1038/NBT.2839

# Supplementary Figures and Tables

## Supplementary Figures


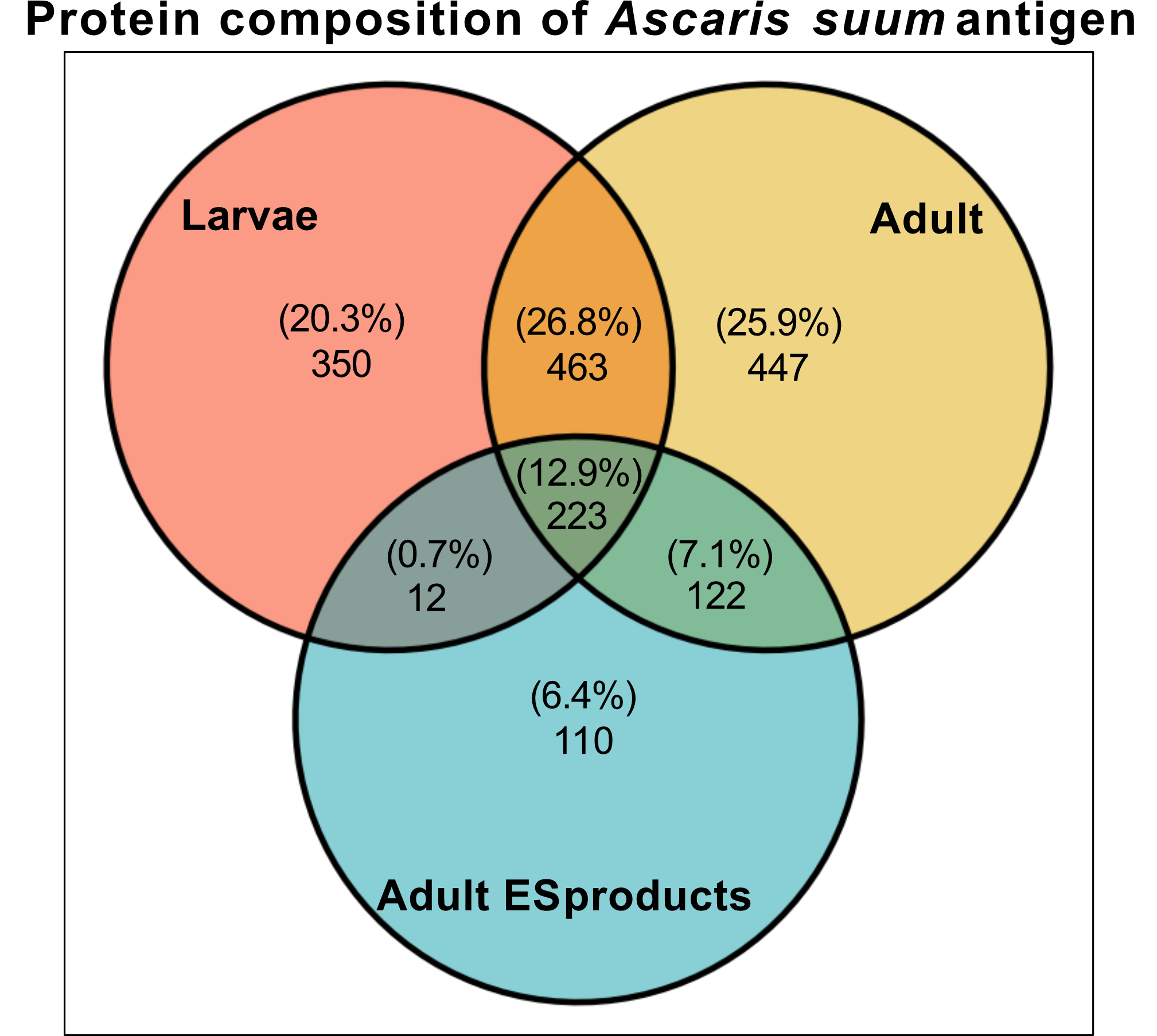


**Supplementary Figure 1. Protein composition of *A. suum* antigens.** Venn diagram depicting the overlap between the somatic (egg-stage L3 and adult) antigens and adult excretory/secretory products (ES).


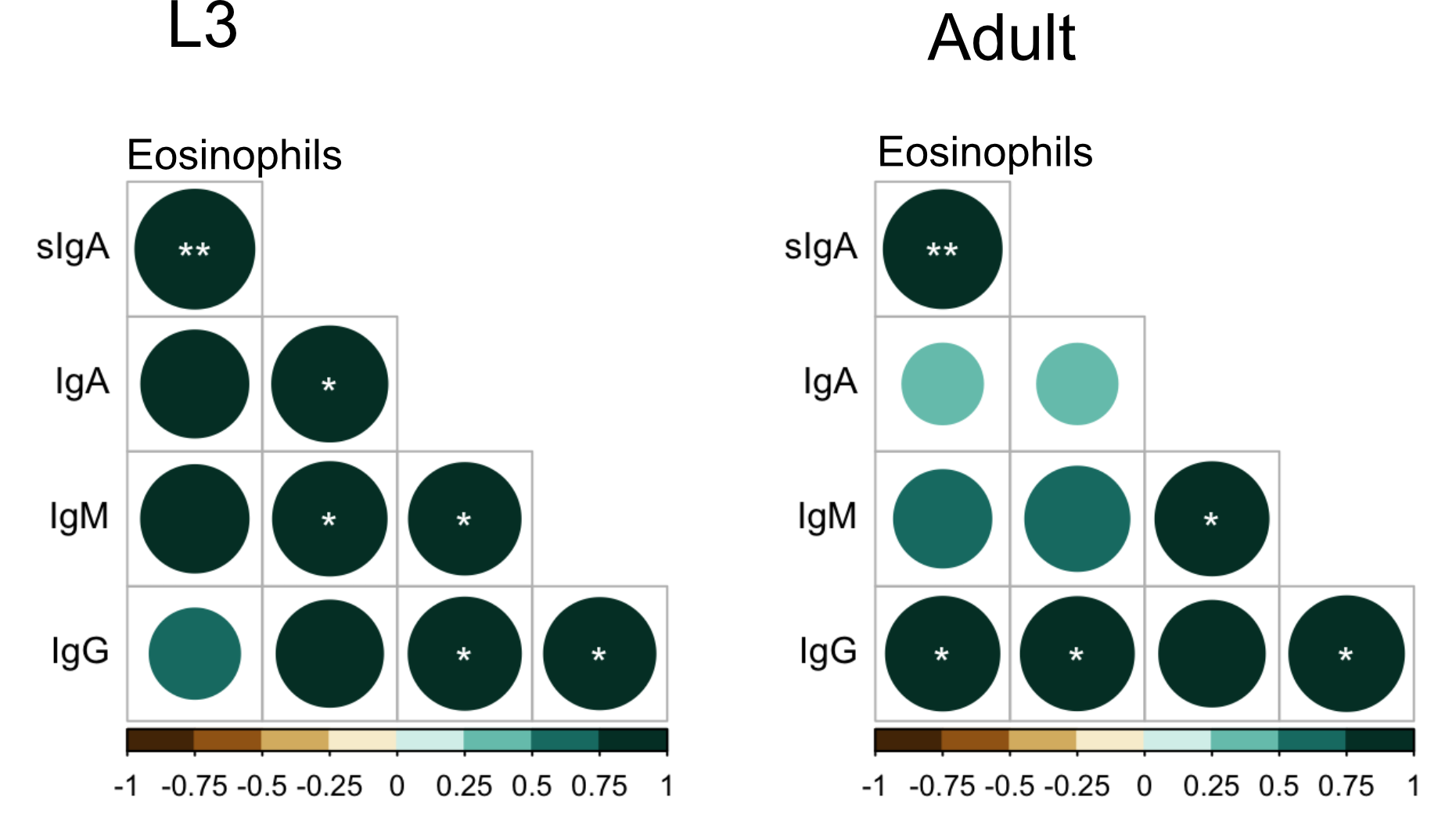


**Supplementary Figure 2. Secretory IgA (sIgA) positively correlates with % eosinophil frequencies of leukocytes in broncho-alveolar lavage (BAL).** Correlation plots depicting the association between eosinophil frequencies and L3- and adult-specific antibody responses in the BAL. Pearson’s correlation was used. Color gradient from brown to blue-green indicate negative to positive correlation. Bubble size increase with increase in correlation coefficient. Significance: p<0.05: *, p<0.005: **.

**
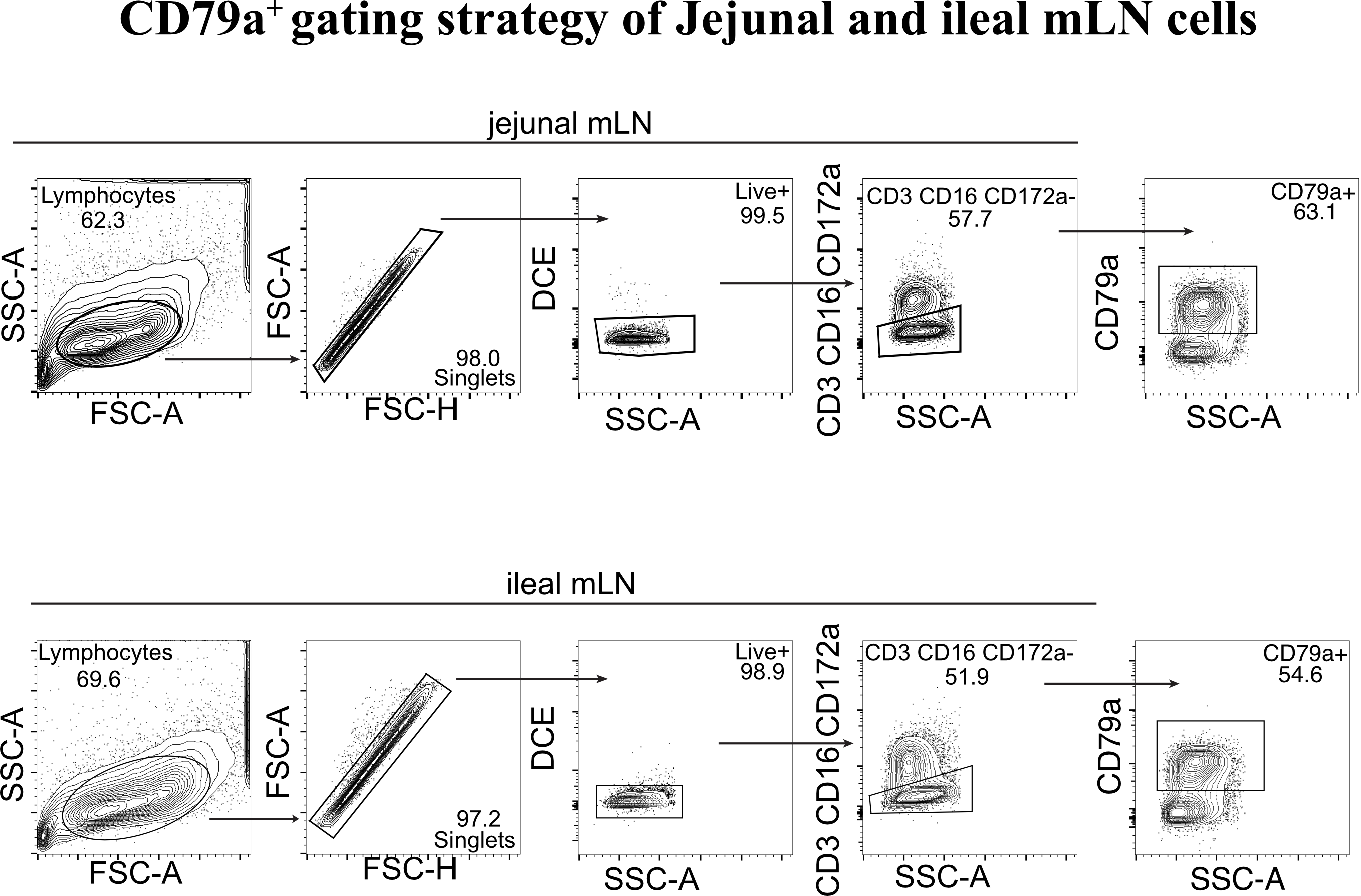
**

**Supplementary Figure 3. Flow cytometry gating strategy.** Representative flow cytometry plots of mLN cells derived from the jejunum and ileum gated on CD79a^+^

## Supplementary Tables

**Supplementary Table 1. Antibodies used for flow cytometry**

| **Target** | **Clone** | **Reactive Species** | **Host Species** | **Isotype** | **Conjugate** | **Company** |
| --- | --- | --- | --- | --- | --- | --- |
| CD3 | BB23-8E6-8C8 | Pig | Mouse | IgG2a | FITC | BD Biosciences |
| CD16 | G7 | Pig | Mouse | IgG1 | FITC | ThermoFisher |
| DCE | - | - | - | - | eFluor 506 | ThermoFisher |
| CD79a | HM47 | Human | Mouse | IgG1 | PE-Vio770 | Miltenyi Biotec |
| IgA | K61 1B4 | Pig | Mouse | IgG1 | Unlabeled | Bio-Rad |
| IgG1 | RMG1-1 | Mouse | Rat | IgG | APC-Cy7 | BioLegend |
| Ki-67 | SolA15 | Dog, Cynomolgus monkey, Human, Mouse, Non-human primate, Rat, pig | Rat | IgG2a, kappa | Alexa Fluor 700 | ThermoFisher |
